# The Role of the Kynurenine Pathway in the Pathophysiology of Frailty, Sarcopenia, and Osteoporosis

**DOI:** 10.3390/nu15143132

**Published:** 2023-07-13

**Authors:** Juan Ballesteros, Daniel Rivas, Gustavo Duque

**Affiliations:** 1 Servicio de Geriatría, Hospital General Universitario Gregorio Marañón, 28007 Madrid, Spain; jubaru4@gmail.com; 2 Research Institute of the McGill University Health Centre, Montreal, QC H4A 3J1, Canada; daniel.rivas@rimuhc.ca; 3 Dr. Joseph Kaufmann Chair in Geriatric Medicine, Faculty of Medicine, McGill University, Montreal, QC H4A 3J1, Canada

**Keywords:** tryptophan, kynurenine pathway, aging, older adults, inflammation, osteoporosis, sarcopenia, frailty, biomarkers, osteosarcopenia

## Abstract

Tryptophan is an essential nutrient required to generate vitamin B3 (niacin), which is mainly involved in energy metabolism and DNA production. Alterations in tryptophan metabolism could have significant effects on aging and musculoskeletal health. The kynurenine pathway, essential in tryptophan catabolism, is modulated by inflammatory factors that are increased in older persons, a process known as inflammaging. Osteoporosis, sarcopenia, osteosarcopenia, and frailty have also been linked with chronically increased levels of inflammatory factors. Due to the disruption of the kynurenine pathway by chronic inflammation and/or changes in the gut microbiota, serum levels of toxic metabolites are increased and are associated with the pathophysiology of those conditions. In contrast, anabolic products of this pathway, such as picolinic acid, have demonstrated a positive effect on skeletal muscle and bone. In addition, physical activity can modulate this pathway by promoting the secretion of anabolic kynurenines. According to the evidence collected, kynurenines could have a promising role as biomarkers for osteoporosis sarcopenia, osteosarcopenia, and frailty in older persons. In addition, some of these metabolites could become important targets for developing new pharmacological treatments for these conditions.

## 1. Introduction

Among the 20 natural amino acids that play a fundamental role in animal nutrition and health, Tryptophan (Trp) must be emphasized. It is a hydrophobic and essential α-amino acid that humans obtain from the diet (i.e., oats, bananas, dried prunes, milk, tuna fish, cheese, bread, chicken, turkey, peanuts, and chocolate), serving as a precursor for several metabolites that play critical roles in body homeostasis [1,2]. Most absorbed Trp is metabolized in the liver, while non-absorbed Trp is metabolized in the gut microbiota [2,3].

Concerning Trp metabolism, the best-known pathway is its conversion to 5-hydroxytryptamine (serotonin), a neurotransmitter mainly involved in controlling adaptive mood and cognition [4]. Since melatonin and N-acetyl serotonin can be produced from serotonin, another role of Trp metabolites is maintaining circadian rhythmicity [5]. However, the kynurenine pathway is the center of Trp degradation [6]. Kynurenines are the main products of this catabolic pathway, involved in several redox reactions, cell differentiation and function, and energy production. According to increasing evidence, the serum levels of kynurenines are independent of Trp intake in the diet. A recent human study found no relation between excessive tryptophan consumption, serum levels of tryptophan metabolites, and bone health [7]. Nevertheless, disturbed Trp catabolism via the kynurenine pathway could significantly impact the pathogenesis of several age-associated conditions, particularly neurodegenerative, autoimmune, and cardiovascular diseases [8,9,10,11,12]. More recently, research efforts have been oriented to evaluate the relationship between the kynurenine pathway and tissue loss/inflammatory age-related conditions such as osteoporosis, sarcopenia, osteosarcopenia, and frailty in older adults with promising results. This review aims to analyze this interplay, gathering the most relevant evidence to date.

### 1.1. Kynurenine Pathway, the Main Route for Trp Metabolism

**Figure 1:**
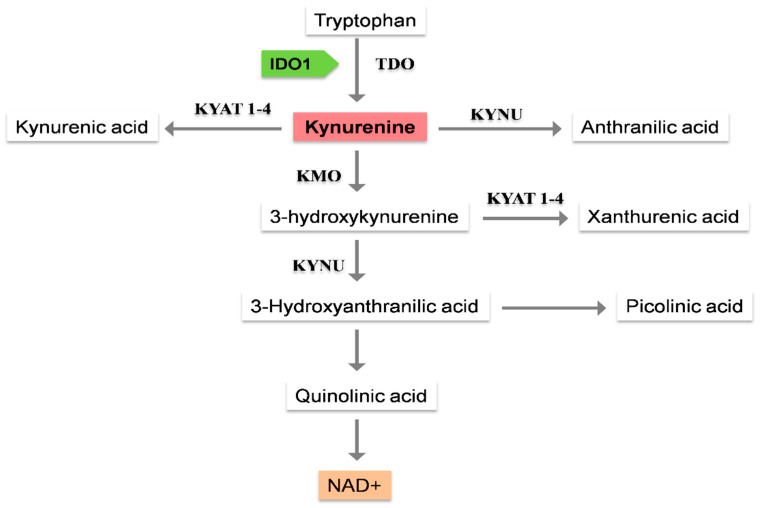
Critical steps of the kynurenine pathway of Trp degradation. Nicotinamide adenine dinucleotide (NAD+), the main end product of this pathway, is a coenzyme for redox reactions central to energy metabolism, which also influences many key cellular functions. Indoleamine 2,3-dioxygenase 1 (IDO1), one of the main enzymes of this pathway, catalyzes the extrahepatic production of kynurenine. Abbreviations: Tryptophan 2,3-dioxygenase (TDO); kynurenine aminotransferase 1-4 (KYAT 1-4); kynurenine 3-monooxygenase (KMO); Kynureninase (KYNU); Kynurenine 3-monooxygenase (KMO).

Kynurenine metabolism is the central catabolic route for ingested Trp [13]. The kynurenine pathway starts with converting Trp into N-formyl kynurenine (NFK). Tryptophan 2,3-dioxygenase (TDO), a tetramer, or indoleamine 2,3-dioxygenase 1 or 2 (IDO1,2), both monomers, are the three enzymes involved in this redox reaction [14,15], which is considered the rate-limiting step for the kynurenine pathway. This reaction is mediated by TDO in the liver [16]. TDO is also the clue determinant of the extrahepatic metabolism of Trp [17,18,19,20]. In this case, the activity of IDO1 and IDO2 enzymes is crucial as potentiators. IDO1 has greater catalytic activity than IDO2, which mainly modulates IDO1’s enzymatic function (Figure 1) [21].

The next step is regulated via mArylformamidaseenzyme (AFMID). AFMID removes the NFK formyl group, which results in obtaining kynurenine (KYN), the main intermediate product of this pathway. KYN can be turned into kynurenic acid (KYNA), anthranilic acid (AA), or 3-hydroxykynurenine (3HK) by kynurenine aminotransferase 1-4 (KYAT 1-4), the kynureninase (KYNU), or the enzyme kynurenine 3-monooxygenase (KMO), respectively (Figure 1) [22]. KYNU and KYAT 1-4 can convert 3HK to 3-hydroxy anthranilic acid (3HAA) or xanthurenic acid (XA). 3HAA can also be produced via a non-enzymatic reaction with AA as the substrate. After a few reactions, 3HAA can be turned into picolinic acid (PIC). As an alternative step, 3-HAA could be quickly converted into quinolinic acid (QUIN) via a non-enzymatic reaction [23]. As the final step of this pathway, NAD+ is produced as the end product of the kynurenine pathway with multiple biological functions associated with energy metabolism, calcium homeostasis, and gene expression [24].

### 1.2. Aging Modulates the Kynurenine Pathway: The Critical Role of IDO1

Numerous studies have shown that aging remodels the immune system and causes senescent non-immune cells to undergo pro-inflammatory alterations [25]. This age-related chronic low-grade inflammation, called inflammaging, can induce significant changes in human tissues [26]. The most significant is the increased expression of inflammatory mediators and the anomalous activation of several signaling pathways [27], including the kynurenine pathway.

**Figure 2:**
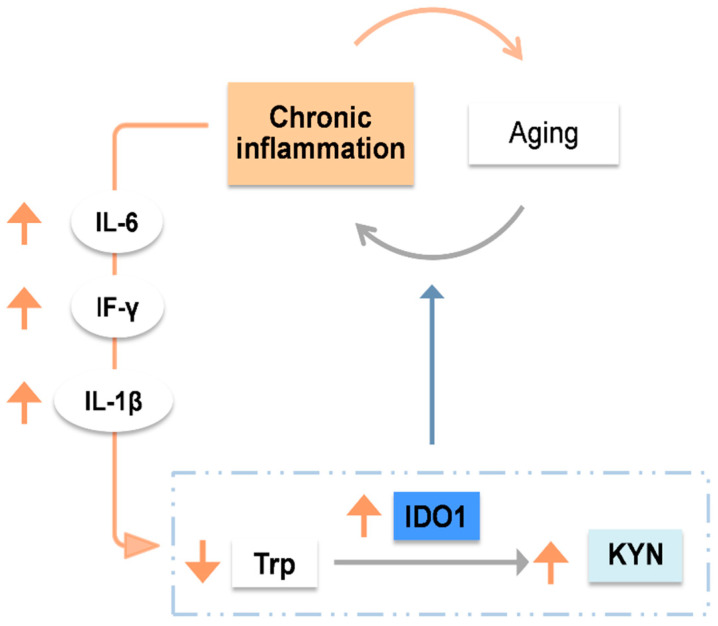
Due to inflammaging, interleukin (IL)-6, IL-1β, and interferon (IF)-γ levels are increased, inducing high levels of indoleamine 2,3-dioxygenase 1 (IDO1) activity. As a result, high kynurenine (KYN) concentrations are observed. This process could explain, in part, the pathogenesis of several tissue loss/inflammatory age-related diseases such as osteoporosis, sarcopenia, osteosarcopenia, and frailty. Trp: tryptophan (Trp).

Aging generates variations in Trp metabolism that are implicated in the pathophysiology of several age-related conditions [12,28]. Indeed, increased IDO1 activation could play a role in the pathogenesis of those disorders [27]. The NF-B and JAK-STAT signaling pathways, which inflammatory factors can activate, can induce the IDO1 gene to express. Levels of KYN are elevated due to the age-related chronic inflammatory stage that induces high IDO1 activity, which is also connected to high levels of interleukin-6 (IL-6), interleukin-1 (IL-1), and interferon γ (IF-γ), all of which are potent activators of IDO1 [12,28,29]. Through this perpetuating cycle of IDO1 activation and inflammaging, Trp depletion and the ensuing buildup of KYN and other catabolic metabolites impair tissue homeostasis and triggers inflammatory diseases. In addition, this chronic process inhibits protein synthesis and promotes host tissue atrophy (Figure 2) [21,30,31,32].

The aryl hydrocarbon receptor (AhR), a potent immunosuppressive transcription factor, has also been involved in this process. Aging is accelerated via increased AhR signaling activity, which also accelerates tissue degradation. Due to the high activity of IDO1 in older persons, particularly those with high levels of inflammaging, high levels of KYN and KYNA can activate the AhR, promoting a breeding ground for chronic age-related diseases [33,34,35,36,37,38,39,40]. In addition, decreased levels of anabolic metabolites, such as PIC and QUIN, have been described in older persons, not only due to inflammaging but also to age-related changes in the gut microbiota [41]

## 2. Kynurenine Pathway: An Unexplored Mechanism for Osteoporosis

### 2.1. The Link between Aging and Osteoporosis

Bone is an active and heterogeneous tissue comprising different cell populations and an extracellular matrix (ECM). Mesenchymal stem cells (MSCs), osteoblasts, osteoclasts, and osteocytes are vital in preserving bone homeostasis and keeping the balance between modeling and remodeling [42,43,44,45,46,47]. In this respect, the differentiation of bone marrow MSCs (BMSCs) into adipocytes and osteoblasts is also crucial [48,49]. The loss of the normal bone microarchitecture, MSCs alterations due to inflammaging, changes in the gut microbiota, and a marked increase in oxidative stress are all mechanisms of age-related bone loss and osteoporosis [42]. This implies a decrease in MSC numbers and a change in their normal function, shifting to more adipogenic differentiation. This complex process, which affects all levels of bone homeostasis, has resulted in bone loss and osteoporosis, where aging is the major risk factor [42,50,51,52,53].

Osteoporosis is characterized by structural bone deterioration and decreased bone mineral density (BMD), frequently associated with frailty, sarcopenia, and falls. In this context, bone fracture risk is increased, feeding this complex circuit that unequivocally implies an increase in functional loss, reduced quality of life, and increased mortality in older persons, making osteoporosis a global problem [54,55,56].

### 2.2. Kynurenine Pathway as a Regulator of Bone Metabolism

**Figure 3:**
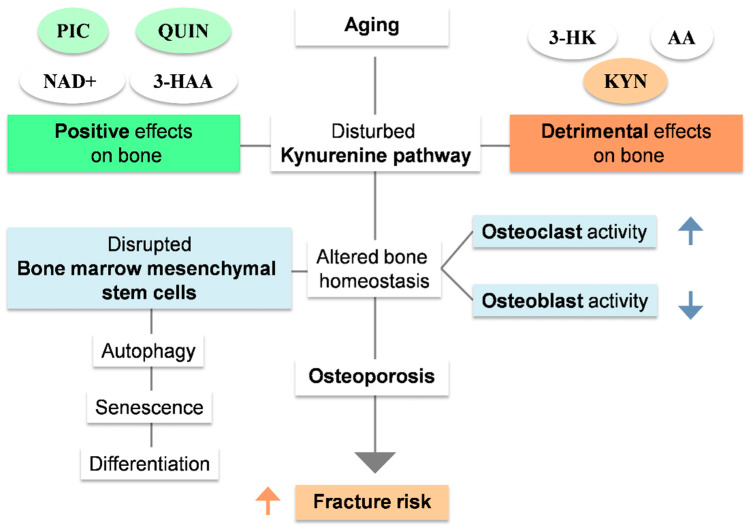
The role of kynurenines in bone metabolism and the pathogenesis of osteoporosis. High levels of kynurenine (KYN), anthranilic acid(AA), and 3-hydroxykynurenine (3-HK) and low levels of quinolinic acid (QUIN), picolinic acid (PIC), 3-hydroxy anthranilic acid (3-HAA), and NAD+ have been associated with changes in bone metabolism and increased risk for osteoporosis and fractures. These changes involved low levels of osteoblast differentiation and function and high levels of marrow adipogenesis and bone resorption by the osteoclasts.

Catabolic Trp metabolites may be critical factors in the development of osteoporosis in older persons (Figure 3). In BMSC, it has been described that several elements of the kynurenine pathway affect osteogenesis and induce dysfunctional autophagy and senescence. KYN decreases CXCL12 protein levels, a critical component of BMSC osteogenesis, via the AhR signaling pathway [57,58,59,60,61]. On the other hand, regarding cellular senescence, KYN increases the level of P21, a cyclin-dependent kinases (CDKs) inhibitor, which forms part of the P21/CDKN1A pathway, thus accelerating senescence changes. Because P53 activates P21, a probable interaction between this tumor-suppressing transcription factor and KYN has been postulated [62,63,64,65,66]. KYN also decreases Hdac3 and CXCL12 via increased miR-29b-1-5p levels, a micro-RNA that downregulates Hdac3 and CXCL12, both osteogenic genes. KYN also upregulates AhR mRNA levels with the deleterious effect described above. The overall effect of such interactions is that BMSCs tend to predominantly adipogenic rather than osteogenic differentiation, which induces a toxic bone marrow microenvironment that increases bone resorption and decreases bone formation, with subsequent bone loss and increased fracture risk [42,67,68,69,70,71,72,73,74].

In addition, strong evidence exists that other kynurenines could also affect bone metabolism [74,75,76,77,78,79,80,81,82,83,84]. High levels of 3-HK and AA have been shown to negatively affect human bone, lowering BMD and increasing fracture risk [75]. 3-HK has a pro-oxidative nature, which can induce ROS leading to cell apoptosis, a phenomenon widely observed in osteoblasts and osteocytes of osteoporotic bone of which its mechanisms are not entirely understood [42,44]. On the contrary, increased BMD and lower fracture risk have been associated with high serum levels of 3-HAA, PIC, QUIN, and NAD+ [74]. 3-AA has been described to have both pro-oxidant and antioxidant effects depending on the context, and the low serum levels observed in patients with osteoporosis may be a response to a lower conversion from AA to 3AA. In addition, xanthurenic acid and 3-hydroxy anthranilic acid were positively associated with BMD among the older participants of the community-based Hordaland Health Study (HUSK) [76]. Regarding KYNA, its effects on bone health have shown divergent results. A study in aged C57BL/6 mice reported that a high-dose KNA decreased trabecular number and thickness as measured via micro-CT [77]. In contrast, a recent study using the same (although younger) mouse model reported that KYNA induces osteogenesis through the Wnt/β-catenin pathway [78]. This evidence suggests that the effect of KYNA on bone cells could depend on age and gender. In contrast, there is solid evidence that PIC has a strong anabolic effect on BMSC both in vitro and in vivo [79,80]. Finally, NAD+, the terminal oxidation product of Trp metabolism, has been reported to alleviate osteoblast senescence and promote bone healing in osteoporotic mice [81].

Regarding kynurenines as indicators of therapeutic response to osteoporosis treatment, Forrest et al. [82] measured serum levels of kynurenines in a cohort of older patients with osteoporosis, observing low serum 3-HAA and high serum AA in that population. Those serum levels were restored to control values after osteoporosis treatment with bisphosphonates. Although these in vitro and in vivo studies suggest a close link between kynurenines and bone health, high-quality studies investigating this role, including the potential administration of anabolic metabolites in humans with osteoporosis and animal models of the disease, are still required. In addition, the effect of current osteoporosis treatments (i.e., denosumab, bone anabolics, etc.) on the kynurenine pathway remains to be investigated.

In summary, current evidence points to the kynurenine pathway as a modulator of MSCs differentiation and bone turnover, making this pathway a potentially useful diagnostic and therapeutic target with prospective future clinical applications in osteoporosis.

## 3. Kynurenines Can Modulate the Risk of Sarcopenia

### 3.1. The Link between Aging and Sarcopenia

The skeletal muscle (SM) makes up between 30 and 40 percent of the weight of an adult person. Its main functions include voluntary movement and regulation of glucose metabolism. Mononucleated myoblasts fuse to create bundles of muscle fibers that make up SM. Moreover, SM is also composed of stem cells known as muscle satellite (stem) cells (MuSCs) and fibro-adipogenic progenitors (FAP). Myoblast fusion, MuSCs, and FAP are essential in SM development and regeneration [84,85,86].

SM is a dynamic organ with plenty of modulators, aging being a critical regulating factor. Aging has been associated with a lower regenerative capacity of SM. Phenotypically this implies a reduced ability to tolerate muscle damage, resulting in muscle atrophy and lost function, clinically known as sarcopenia [87,88]. Numerous studies have documented myofiber nuclei loss, decreased myofiber number, and adipose and fibrotic tissue infiltration in sarcopenia. In this respect, inflammaging and age-related oxidative stress age-related increase the activation of several cell signaling pathways such as WNT, TGF-b-, FGF2, JAK/STAT3, p38 and p16^INK4a^, which can reduce MuSCs regeneration capacity and induce them into a mainly pre-senescent status or apoptosis, while also generating a predominantly adipogenic differentiation of the FAP [89,90,91,92,93,94,95,96,97,98,99].

Low strength, reduced muscle mass, and poor physical performance are characteristics of sarcopenia. Its incidence rises with age and is especially linked with the likelihood of hospitalization in old patients and increased morbidity and frailty [100,101,102,103,104]. Sarcopenia has also been associated with increased risk of fractures and falls, mortality, and health care costs. When concomitantly occurring with osteoporosis, it configures a syndrome known as osteosarcopenia [105,106,107,108]. To date, there is not an international consensus about sarcopenia, especially in the diagnosis and pharmacological treatment, making this entity a promising target for studies.

### 3.2. The Role of Kynurenines in Sarcopenia: A Promising Interplay

**Figure 4:**
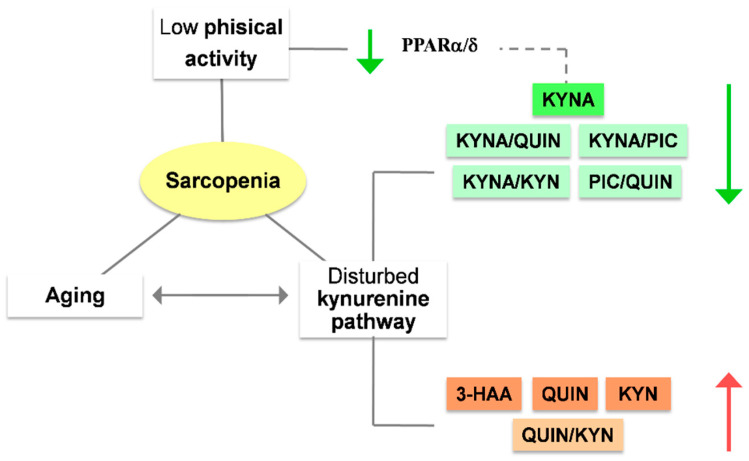
Sarcopenia has been linked with high levels of 3-HAA, QUIN, KYN, and QUIN/KYN ratios, as well as low levels of KYNA and KYNA/QUIN, KYNA/PIC, KYNA/KYN, and PIC/QUIN ratios. Low physical activity, a major risk factor for sarcopenia, can modulate the kynurenine pathway with less KYN conversion to KYNA due to low PPARα/δ. Aging can also affect the kynurenine pathway and induce sarcopenia via inflammaging. Abbreviations: Kynurenic acid (KYNA); Kynurenine (KYN); Quinolinic acid (QUIN); Picolinic acid (PIC); 3-hydroxy anthranilic acid (3-HAA).

A connection between sarcopenia and the kynurenine pathway has been reported (Figure 4). This pathway likely contributes to the atrophy of SM and oxidative stress via KYN, which is increased with the high levels of inflammaging observed in sarcopenia. Some studies have demonstrated that KYN administration decreases muscle power and size while increasing muscle lipid peroxidation, protein catabolism, and reactive oxygen species (ROS) levels independently of Ahr activation [109,110]. Chronically elevated ROS levels and increased lipid peroxidation products, all induced by high levels of KYN, have been associated with sarcopenia [111,112]. In contrast, it has been shown that inhibiting IDO1 activity results in a bigger and stronger SM [109,110]. 

Furthermore, physical exercise is another SM modulator that affects strength and function and regulates nutrient metabolism. Aerobic exercise increases human lean muscle levels of PGC-1α1, a transcriptional coactivator essential for adaptive tissue response [113]. Activated PGC-1α1 together with PPARα/δ increases *KAT* gene expression in SM, reducing KYN levels and increasing KYNA production [114,115].

Studies that looked at the connection between Trp metabolites and physical function in aged people found that KYNA levels, as well as KYNA/KYN, PIC/QUIN, KYNA/PIC, and KYNA/QUIN ratios, were linked to less sarcopenia, measured with grip strength and gait speed parameters. Lower physical function, on the other hand, led to higher concentrations of 3-HAA, QUIN/KYN ratio, and QUIN. Additionally, those with increased levels of KYN, KYN/TRP, 3-HK, QUIN, and QUIN/KYN ratios had elevated IL-6 levels in serum (Figure 4) [116].

Overall, kynurenines can be considered biomarkers and potential therapeutic targets for sarcopenia in the future. However, despite a clear biological plausibility, further investigating the underlying mechanisms is needed to allow the use of kynurenine pathway metabolites in developing new diagnostic and therapeutic strategies to tackle sarcopenia and osteosarcopenia.

## 4. Frailty, an Expression of Aging: Do the Kynurenines Have Something to Tell?

Frailty is a clinical condition defined as impaired strength, endurance, and physiologic reserve in older persons [117,118,119,120]. It is diagnosed in clinical practice using well-validated criteria, including five clinical criteria proposed by Fried et al. [118] or deficit accumulation proposed by Rockwood et al. [119]. Due to its multidimensional nature, frailty origin responds to the interaction of physical, biological, psychological, genetic, environmental, and social factors [117,118,119,120]. Frailty increases the body’s susceptibility to acute illness and stressors, raising the risk for disability, institutionalization, falls, fractures, and mortality [121,122]. These features are somewhat reminiscent of osteosarcopenia. In fact, it has been proposed that frailty and osteosarcopenia are part of a clinical spectrum with similar pathophysiology [106].

**Figure 5:**
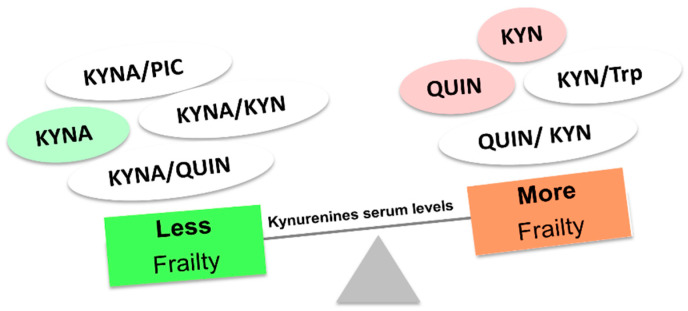
Serum levels of kynurenines are altered in frail older persons. Abbreviations: Kynurenic acid (KYNA); Kynurenine (KYN); Quinolinic acid (QUIN); Picolinic acid (PIC); tryptophan (Trp).

The reduction in physiological reserve observed in frailty has been closely associated with inflammaging [123]. For this reason, it is challenging to present these two entities as independent processes, particularly in the context of the kynurenine pathway. Indeed, several studies have linked physical frailty, frailty index, and some physical parameters with some components of the kynurenine pathway. High KYN/Trp ratio levels have been associated with frailty in older adults diagnosed according to Fried’s criteria [121,123,124]. Along the same line, high levels of KYN have been seen in the serum of frail patients diagnosed using both Fried and Rockwood criteria, also obtaining significant associations by analyzing KYN interaction with physical parameters, including time to complete five chair stands, grip strength, and gait speed [125]. In another sample of older persons, low KYNA, KYNA/KYN, KYNA/QUIN, and KYNA/PIC ratios were linked to a decreased chance of being frail according to Fried’s and Rockwood’s criteria. On the other hand, QUIN and QUIN/KYN ratios showed an association with a higher probability of frailty and increased IL-6 levels. Regarding physical parameters, a reduction in gait speed was observed at high levels of 3-HAA and QUIN. Less grip strength was also associated with a high QUIN/KYN ratio, whereas better grip strength and gait speed were seen in patients with a higher KYNA/QUIN ratio (Figure 5) [116].

In summary, frailty is a complex entity, which makes developing biomarkers and treatments extremely challenging. These collected evidence suggest that kynurenines could be an attractive biomarker for assessing frailty in humans. Moreover, due to its osteogenic and myogenic effect in vitro and in vivo, some anabolic metabolites of the kynurenine pathway (i.e., PIC or NAD+) could have therapeutic potential for frailty. However, further evidence is still needed.

## 5. Conclusions

In this comprehensive literature review, we have summarized most of the current evidence on the roles that several elements of the kynurenine pathway have on the formation, metabolism, and function of muscle and bone. Some pathway elements such as KYN, KYNA, and QUIN have been associated with a deleterious effect on muscle and bone formation and function and with the pathogenesis of osteoporosis, sarcopenia, osteosarcopenia, and frailty. On the other hand, some anabolic metabolites such as PIC and NAD+ have a beneficial effect on muscle and bone with the potential to become novel therapeutic approaches for those conditions.

The kynurenine pathway regulates multiple functions associated with bone and muscle biology. Several elements of this pathway are involved in the differentiation of osteoblasts and myoblasts and, thus, bone and muscle formation, respectively. Those metabolites regulate vital biological processes involved in the differentiation and function of BMSC and mature osteoblasts and myocytes, such as autophagy and apoptosis. Finally, several elements of this pathway are closely involved with regulating senescence in these cells. Taken together, solid in vitro and in vivo evidence supports a strong biological connection between multiple elements of the kynurenine pathway and muscle and bone health with strong potential to be translated into new biomarkers and therapies for several age-related conditions, particularly those associated with inflammaging such as sarcopenia, osteoporosis, osteosarcopenia, and frailty.

From a clinical perspective, associations between several elements of the kynurenine pathway (including their ratios) have been cross-sectionally assessed, demonstrating a biological plausibility between changes in serum concentrations of several metabolites and the occurrence of osteoporosis, sarcopenia, and frailty. Interestingly, these associations seem to be independent of the amount of Trp in the diet, thus suggesting that alterations in Trp metabolism induced by inflammaging and/or gut microbiota could explain those changes in serum levels of potentially toxic metabolites opening an interesting field of research to determine the value of these metabolites as novel diagnostic and therapeutic approaches to these conditions in humans. Finally, preliminary data suggest that treating osteoporosis could also affect Trp metabolites associated with bone metabolism, opening a potential role as biomarkers of this and other age-related musculoskeletal conditions.

## 6. Future Directions

Research on the kynurenine pathway’s role in osteoporosis is still in its early stages. More research is required to fully comprehend how kynurenines affect bone metabolism and contribute to the onset of osteoporosis. This involves analyzing kynurenines’ effects on bone cells and their interaction with signaling pathways associated with bone homeostasis. The future use of kynurenines as diagnostic biomarkers might help with early identification and risk assessment for osteoporosis, enabling focused treatments and tracking treatment effectiveness. Therapies targeting the kynurenine pathway’s enzymes or products might stop bone loss and stimulate bone growth. Before being used in clinical settings, these therapies’ safety and effectiveness must be extensively examined in preclinical studies. Insights into the relationship between kynurenines and osteoporosis can be gained via large-scale clinical trials and population-based research, including exploring interventions that target the kynurenine pathway in conjunction with already available therapies to test their synergistic benefits.

Regarding sarcopenia, exercise, and resistance training can stop or slow the progression of sarcopenia. Adequate protein intake and dietary interventions are crucial for maintaining muscle health. There is still no medication available for preserving muscle mass and functionality. In this respect, identifying kynurenines with anabolic effects on SM may provide new molecules for tackling sarcopenia. Moreover, considering that exercise has a beneficial impact on the kynurenine pathway, studies combining therapeutic approaches, such as exercise, nutrition, and these Trp metabolites could provide better outcomes for older persons with sarcopenia. Additionally, biomarkers for sarcopenia monitoring and early diagnosis are being sought, and the potential use of kynurenines as biomarkers for sarcopenia could be revealed in the future. However, to obtain reliable information about using kynurenines in sarcopenia assessment and treatment, more research and understanding of this entity in older adults is still needed.

Finally, investigating the kynurenine pathway metabolites may shed light on the underlying mechanisms of frailty and identify new targets for diagnosing and treating this condition. Population-based studies with a cohort of robust older adults that evaluate the incidence of frailty while monitoring kynurenine levels may give us information about the use of these metabolites in the early diagnosis of frailty and their predictive value for adverse outcomes in this population.

Overall, the kynurenine pathway is one of the connecting mechanisms between sarcopenia, osteoporosis, osteosarcopenia, and frailty. This close connection could be the source of future combined biomarkers and novel therapeutic approaches for these conditions that deserve further investigation.

## Data Availability

Not applicable.
